# Toxic Mechanism and Biological Detoxification of Fumonisins

**DOI:** 10.3390/toxins14030182

**Published:** 2022-03-01

**Authors:** Linkai Qu, Lei Wang, Hao Ji, Yimeng Fang, Pengyu Lei, Xingxing Zhang, Libo Jin, Da Sun, Hao Dong

**Affiliations:** 1 College of Life Sciences, Jilin Agricultural University, Changchun 130118, China; qulinkai@wzu.edu.cn; 2 Biomedical Collaborative Innovation Center of Zhejiang Province, Institute of Life Sciences, Wenzhou University, Wenzhou 325035, China; wanglei07210228@163.com (L.W.); joe323@wzu.edu.cn (H.J.); fym15088993352@163.com (Y.F.); leipengyu0411@163.com (P.L.); libo9518@126.com (L.J.); 3 Department of Endocrinology and Metabolism, The First Affiliated Hospital of Wenzhou Medical University, Wenzhou 325000, China; zhangxingxing@wzhospital.cn

**Keywords:** fumonisins, toxicity mechanism, toxins structure, biological detoxification, antioxidants detoxify

## Abstract

Food safety is related to the national economy and people’s livelihood. Fumonisins are widely found in animal feed, feed raw materials, and human food. This can not only cause economic losses in animal husbandry but can also have carcinogenicity or teratogenicity and can be left in animal meat, eggs, and milk which may enter the human body and pose a serious threat to human health. Although there are many strategies to prevent fumonisins from entering the food chain, the traditional physical and chemical methods of mycotoxin removal have some disadvantages, such as an unstable effect, large nutrient loss, impact on the palatability of feed, and difficulty in mass production. As a safe, efficient, and environmentally friendly detoxification technology, biological detoxification attracts more and more attention from researchers and is gradually becoming an accepted technique. This work summarizes the toxic mechanism of fumonisins and highlights the advances of fumonisins in the detoxification of biological antioxidants, antagonistic microorganisms, and degradation mechanisms. Finally, the future challenges and focus of the biological control and degradation of fumonisins are discussed.

## 1. Introduction

Mycotoxins are toxic secondary metabolites produced by many filamentous fungi of ascomycetes [1]. Mycotoxin pollution is a persistent global problem which is inevitable and unpredictable. The production of mycotoxins is affected by the surrounding environment; even a good growth and storage environment cannot completely prevent the production of mycotoxins [2]. Fumonisins are a group of toxins that pose a significant threat to food and animal health after aflatoxins. Fumonisins have high toxicity and often appear together with aflatoxin toxicity. They cause huge economic losses to the livestock and poultry breeding industry and threaten human health [3,4]. Therefore, several studies have been exploring methods to control and alleviate fumonisin toxicity. Fumonisins easily contaminate corn, rice, and other grains, causing damage to the liver and kidneys of several animals that feed on these grains and even causing tumor problems [5,6]. In addition, fumonisin toxicity is implicated in causing human esophageal cancer and neural tube defect disease [7,8], thus fumonisins have gradually become a research hotspot after aflatoxin.

Fumonisins are a water-soluble secondary metabolite mainly produced by *Fusarium verticillioides*, *Fusarium proliferatum,* and other *Fusarium* species [9]. It exists on a variety of substrates, mainly on grains such as corn, and can also be found in products manufactured using grains as raw materials [5]. Fumonisins can be divided into four categories: A, B, C and P, including 28 structural analogues: FA_1,_ FA_2,_ FA_3,_ PHFA_3a,_ PHFA_3b,_ HFA_3,_ FAK_1,_ FBK_1,_ FB_1,_ Iso-FB_1,_ PHFB_1a,_ PHFB_1b,_ HFB_1,_ FB_2,_ FB_3,_ FB_4,_ FB_5,_ FC_1,_ N-acetyl-FC_1,_ Iso-FC_1,_ N-acetyl-iso-FC_1,_ OH-FC_1,_ N-acetyl-OH-FC_1,_ FC_3,_ FC_4,_ FP_1,_ FP_2_, and FP_3_ (Table 1). Notably, the fumonisin B family is the main and most toxic family. Fumonisin B_1_ (FB_1_) and fumonisin B_2_ (FB_2_) are the most abundant and most toxic variants that naturally contaminate maize, accounting for 70–80% and 15–25% of the total number of fumonisins [10,11].

WHO (2001) established a provisional maximum daily tolerable level of fumonisins at 2 μg/kg-BW (body weight), owing to its high levels and high toxicity [12]. The European Commission (2006 and 2007) set the maximum levels of fumonisins for unprocessed maize at 4000 μg/kg, FB at 1000 μg/kg for human corn-based foods, 800 μg/kg for corn breakfast cereals and snacks, and 200 μg/kg for corn-based baby foods [13,14]. The International Agency for Research on Cancer (IARC) classifies fumonisins into group 2B, which is a possible human carcinogen owing to their harmful effects [15]. Therefore, it is particularly significant to reduce the content and detoxify fumonisins in food.

Fumonisins are highly soluble in water and have strong thermal stability, thus they are chemically stable under various conditions. It is therefore challenging to remove them from ordinary grain processing to meet normal edible standards [16]. Physical and chemical methods cannot effectively remove fumonisins and other toxic substances from grains. Studies report that biological methods can effectively remove fumonisins in crops. Therefore, studies have widely explored the inhibition of fumonisin-producing strain growth and degradation of fumonisins through biological control and biodegradation [17,18].

In the current paper, the current prevention and control methods of fumonisin-producing strains, microorganisms that can degrade fumonisins, and biodetoxification methods were reviewed. This work will provide a reference for the biological control of fumonisin-producing strains and information on biodegradable fumonisin-producing strains.

## 2. Toxic Mechanism of Fumonisins

Fumonisins cause a variety of toxic effects to organisms including autophagy, apoptosis, neurotoxicity, immunotoxicity, reproductive toxicity, tissue and organ toxicity, and carcinogenicity. They can not only cause disease alone but also have a combined toxic effect with other mycotoxins such as aflatoxins. The toxicity of fumonisins is a very complex process. Previous studies report that fumonisins exert their toxicity by modulating sphingolipid metabolism and inducing oxidative stress [19,20].

### 2.1. Effect of Fumonisins on Sphingolipid Synthesis

**Figure 1:**
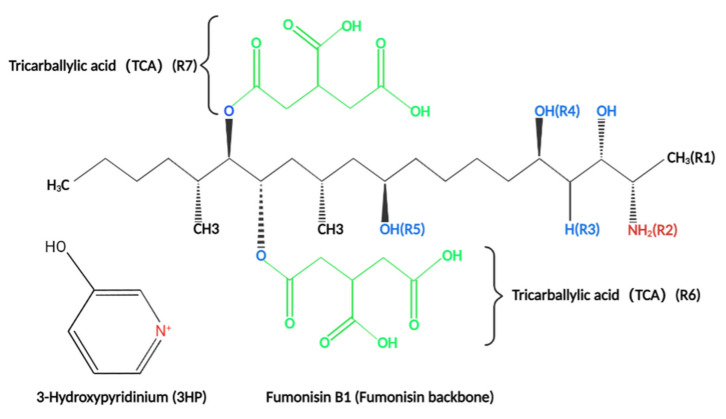
The basic structure of fumonisins and the structural groups of fumonisin analogs (fumonisins analogs and R side chain structures are presented in Table 1). Fumonisins have a basic skeleton composed of 20 carbons and various carboxyl groups; hydroxyl groups, as well as ester bonds, are distributed on both sides of the skeleton.

**Figure 2:**
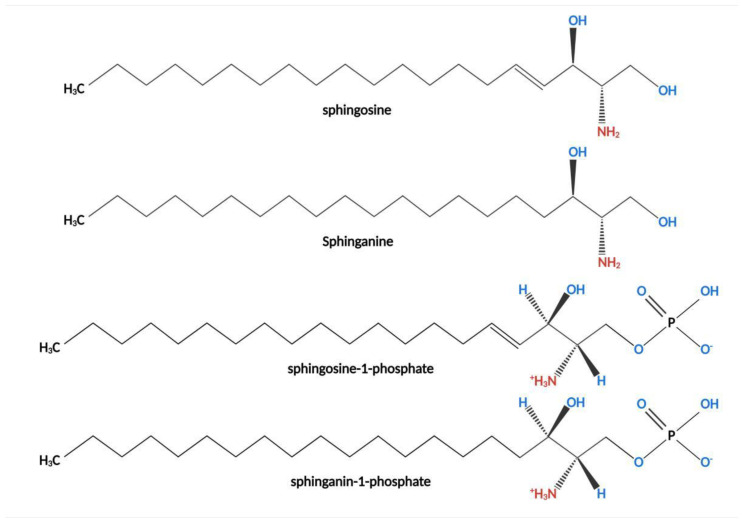
Structure of Sphingosine, Sphinganine, sphingosine-1-phosphate, and sphinganin-1-phosphate. Sphingosine and Sphinganine are the key reactants in the synthesis of ceramide and participate in the composition of the membrane structure. The structures are highly similar to the structures of fumonisins, with a long chain skeleton and amino groups on the side of the skeleton.

Fumonisins (such as FB_1_) are a class of structurally similar diesters comprising different polyols and glycerol tricarboxylic acids (Figure 1). They have a similar structure to that of sphingosine (So) (Figure 2) and sphinganine (Sa) (Figure 2). Therefore, they are classified under sphingosine-like mycotoxins [21,22]. So and Sa are the main components of sphingomyelin. Sphingolipid is an important component of biofilm. Sphingolipid is involved in the regulation of several signal transduction processes such as cell proliferation, differentiation, senescence, apoptosis, and carcinogenesis. Notably, sphingolipid is the key hub of cell-to-cell recognition and interaction [23,24,25].

**Figure 3:**
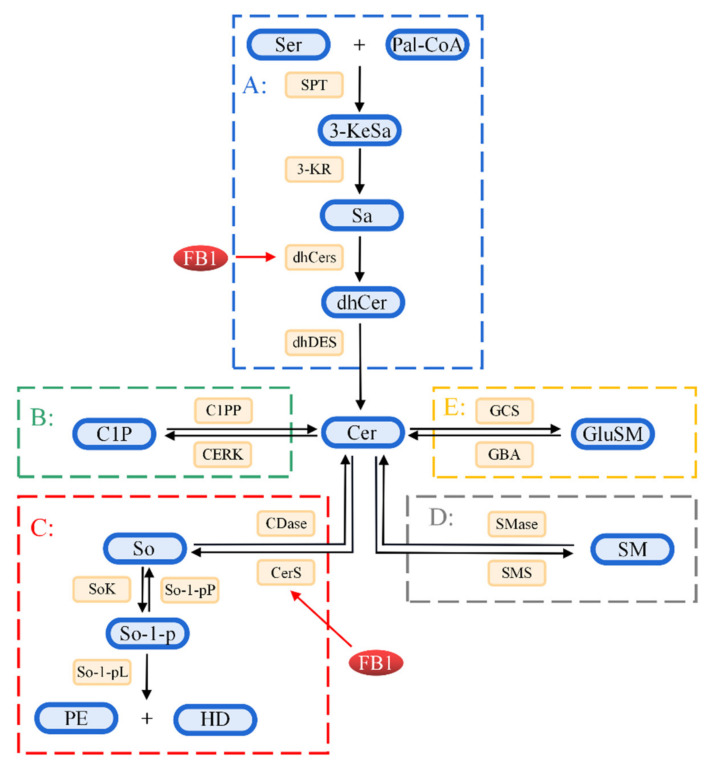
Schematic illustration of sphingolipid metabolism. A: The synthetic pathway of ceramide. Serine (Ser) and palmitoyl coenzyme A are condensed under the action of the serine palmitoyltransferase (SPT) enzyme to form 3-keto-Sphinganine (3-KeSa). 3-KeSO is reduced under the action of 3-keto reductase (3-KR) to produce Sphinganine (Sa). Further, Sa is converted to dihydroceramide (dhCer) under the action of dihydroceramide synthetase (dhCerS)/ceramide synthetase (CerS). Finally, dhCer produces ceramide (Cer) under the action of dihydroceramide dehydrogenase (dhDES). B, C: The circulation pathway. Ceramide is phosphorylated to produce ceramide-1-phosphate (C1P) under the action of ceramide kinase (CERK). C1P can also be phosphorylated to form Cer under the action of C1P phosphatase (C1PP). In addition, ceramide produces sphingosine (So) under the action of ceramidase (CDases), which is a reversible reaction, whereby Cer can be formed under the action of CerS. Generated So is phosphorylated to form sphingosine-1-phosphate (So-1-p) under the action of sphingosine kinase (SoKs). So-1-p is converted to So under the action of sphingosine-1-phosphate phosphatase (So-1-pP). So-1-p is pyrolyzed to phosphoethanolamine (PE) and hexadecanal (HE) under the action of So-1-p lyase (So-1-pL). D, E: The hydrolysis pathway. In the hydrolysis pathway, sphingomyelin (SM) is hydrolyzed by sphingomyelinase (SMases) to form Cer and phosphocholine, whereas Cer is catalyzed by sphingomyelin synthase (SMSs) to form SM and diacylglycerol. Glycosphingolipids (GlySM) produce Cer under the action of glucerebrosidase (GBA). Further, Cer produces glycosphingolipids under the action of glucosyl-ceramide synthase or galactosyl-ceramide synthase (GCS).

So and Sa are biosynthesized as condensation between palmitoyl-COA and serine as substrates under the actions of serine palmitoyltransferase, ketoreductase, dihydroceramide synthetase, dihydroceramide dehydrogenase, and other enzymes under normal physiological conditions. FB_1_ competes and inhibits ceramide synthetase owing to the similar structure to that of So and Sa (Figure 3). Ceramide can be produced through ab initio synthesis, or through sphingomyelin hydrolysis and sphingomyelin circulation, which is the central link of sphingomyelin metabolism. Dysregulation of ceramide synthesis affects sphingomyelin metabolism (Figure 3) [22,23], resulting in damage to the integrity of the cell membrane. Accumulation of phosphorylated products of So, Sa and So, Sa, sphingosine-1-phosphate (So-1-p) (Figure 2), and sphinganin-1-phosphate (Sa-1-p) (Figure 2) in cells can also cause cellular dysfunction [19].

The contents of Sa and So in blood and cerebrospinal fluid of pigs were significantly higher compared with those in the control group after intravenous administration of 139 nmol of FB_1_ or oral administration of 3425 nmol/kg-BW FB_1_ [21], therefore, Sa and So are biomarkers of FB_1_ exposure in vivo. Waes et al. reported that the content of Sa-1-p in plasma of LM/Bc mice treated with 40 μM FB_1_ was significantly higher relative to that of the control group [26], This dose of FB_1_ increases the rate of embryonic malformation and the risk of neural tube defects in pregnant LM/BC mice [27]. Moreover, Kim et al. reported that accumulation of Sa-1-p and So-1-p in serum was significant compared with that of Sa and So after intraperitoneal administration of 10 mg/kg FB_1_ in mice for five consecutive days [28]. In addition, the kidney is the main metabolic organ of FB_1_; Sa-1-p, and So-1-p produced by FB_1_ metabolism accumulate and last longer in the kidney relative to FB_1_, and So-1-p and Sa-1-p in cells can cause cellular dysfunction, implying that Sa-1-p and So-1-p can also be used as biomarkers for FB_1_ exposure in the body. This explains why the kidney is more vulnerable to injury compared with other organs [29,30]. Grenier et al. reported that fumonisins cause changes in the contents of Sa and So and exert a significant increase in levels of pro-inflammatory factors and Th1/Th7 in the small intestine with the increase in fumonisin concentration and feeding time [31]. Bracarense et al. reported similar findings when studying the effect of FB_1_ on the expression of pro-inflammatory cytokines mRNA in porcine jejunal epithelial cells. They found that the expression of IFN- γ and IL-10 in porcine jejunal epithelial cells increased significantly [32]. These findings indicate that FB_1_ affects sphingolipid metabolism and exerts cytotoxicity by modulating the expression of proinflammatory cytokines.

### 2.2. Fumonism Induces Oxidative Stress

Oxidants and antioxidants in the body are in a state of dynamic balance under physiological conditions. The body produces potentially excessive toxic aerobic free radicals, on exposure to FB_1_, which cannot be neutralized by antioxidants present in cells. High levels of aerobic free radicals result in lipid peroxidation, DNA oxidative damage, decreased glutathione (GSH) content, and the down-regulated expression of glutathione peroxidase (GPx) and superoxide dismutase (SOD), ultimately leading to cell tissue damage and dysfunction [33,34,35,36,37,38].

Studies report that FB_1_ mediates cytotoxicity partially through the induction of oxidative stress. For example, treatment of HepG2 cells with 50 μM FB_1_ for 0, 12, and 24 h, significantly increased the ROS content in HepG2 cells treated with FB_1_ compared with the level of the control group [38]. The levels of SOD-1, SOD-2, glutathione reductase (GR), and catalase (CAT) in the colon tissue of mice exposed to 2.5 mg/kg-BW FB_1_ for 4 consecutive days were significantly lower relative to the levels of the control group. On the contrary, expression levels of CYP450, thioredoxin, heat shock protein 70, and heat shock protein 90 were significantly higher relative to the expression levels of the control group [20]. Exposure of human SH-SY5Y neuroblastoma, rat C6 glioblastoma, and mouse GT1-7 hypothalamic cells to 0.1–100 μM FB_1_ for 0–144 h induced C6 and GT1-7 ROS formation in a dose-dependent manner, however, it had no significant effect on SH-SY5Y. Moreover, it downregulated GSH expression, increased malondialdehyde (MDA) production, and promoted lipid peroxidation and necrotic cell death in all cells [36].

In addition to oxidative stress, FB_1_ can cause damage to cell DNA. Domijan et al. observed that the apoptosis rate was significantly increased in adult male rats 48 h after administration of 5 μg/kg-BW FB_1_, in a dose-dependent manner. Furthermore, treatment of adult male rats with 500 μg/kg-BW FB_1_ induced significant DNA damage [39]. DNA damage is the basis of cell carcinogenesis. Treatment of frozen horse spermatozoa with 2.5 × 10^−5^ μM FB_1_ showed significant damage to sperm chromosomes resulting in reproductive toxicity [40]. Exposure of C6 glioma cells and p53 deleted mouse embryonic fibroblasts to FB_1_ significantly increased the content of MDA in C6 glioma cells, increased the apoptotic rate of C6 glioma cells, and increased the levels of 8-OH-dG and DNA fragments in C6 glioma cells in a dose-dependent manner [41]. 8-OH-dG is an important marker for DNA oxidative damage and cell carcinogenesis [42,43]. This finding indicates that FB_1_ leads to DNA oxidative damage. In addition, Yuan et al. reported that exposure of pig iliac endothelial cells to 50 µg/mL FB_1_ induced a significant increase in intracellular MDA content and a decrease in SOD, CAT, and GSH levels. Moreover, the findings showed that FB_1_ affects the expression of porcine vascular endothelial cells’ tight junction proteins [44]. Yu et al. conducted a subsequent study and reported that FB_1_ promotes cell proliferation and migration as well as induces carcinogenesis of human esophageal epithelial cells. Notably, FB_1_ significantly upregulates the expression of cell cycle regulatory proteins (cyclinD1 and cyclinD3) and downregulates the expression of tumor suppressor genes such as phosphatase, tensin homolog, and adenomatous polyposis, indicating that FB_1_ may exert its toxic or carcinogenic effects by modulating the cell cycle [45].

**Figure 4:**
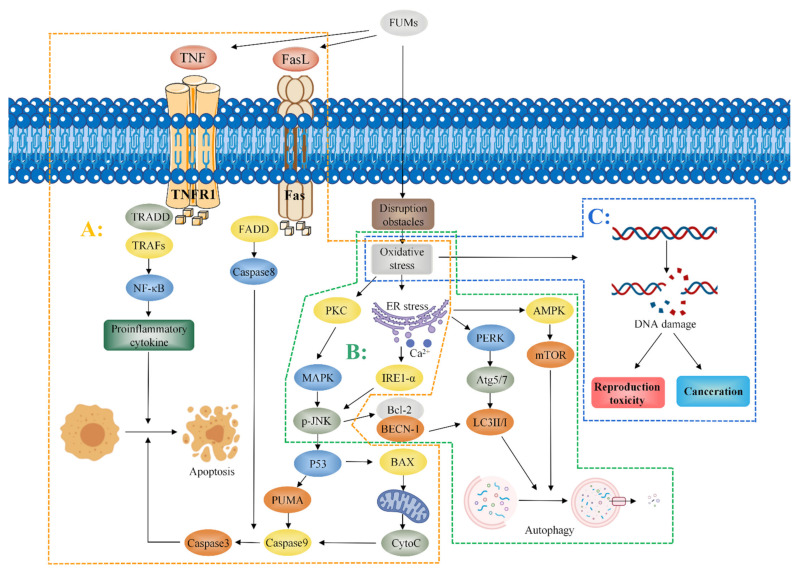
Mechanism of fumonisin toxicity. Fumonisins induce oxidative stress and mediate apoptosis, autophagy, reproductive toxicity, and promote carcinogenesis. A: The apoptosis pathway. FB1 activates PKC, which activates the JNK signaling pathway through MAPK. Meanwhile, FB_1_ induces oxidative stress, resulting in the release of Ca^2+^ from the endoplasmic reticulum; increased Ca^2+^ release leads to phosphorylation of JNK, which further activates the P53 apoptosis signaling pathway. P53 up-regulates the expressions of pro-apoptotic factors PUMA and Caspase3 and activates BAX, which induces increased CytoC expression. It promotes the expression of Caspase9 and Caspase3 and induces apoptosis. FB_1_ also leads to Fas receptor dysfunction, which activates Caspase8, and Caspase8 induces increased expression of Caspase3, leading to apoptosis; FB_1_ induces apoptosis by activating the TNF-α signaling pathway and upregulating NF-κB, an important target of the TNF-α signaling pathway. B: The autophagy pathway. FB_1_ mediates the JNK pathway through oxidative stress, promotes the interaction between BAL-2 and BECN-1, and induces increased LC3-II/I expression. Meanwhile, FB_1_ mediates PERK through the endoplasmic reticulum, induces ATG5/7 expression, increases LC3-II/I expression, and induces apoptosis. FB_1_ also up-regulates the AMP-dependent AMPK expression through endoplasmic reticulum stress and increased AMPK expression down-regulates the mTOR induced autophagy. C: Reproductive toxicity and carcinogenesis. Oxidative stress induces DNA damage which in turn induces reproductive toxicity and carcinogenesis.

Oxidative stress induced by FB_1_ partially mediates apoptosis and autophagy. Mitogen-activated protein kinase (MAPK) is an important messenger in cells. When FB_1_ is administered to cells, it activates protein kinase C (PKC) and regulates the c-Jun N-terminal kinase (JNK) signal pathway through MAPK [46,47]. Administration of 2.5 mg/kg-BW FB_1_ to mice for 4 days induced oxidative stress and the endoplasmic reticulum release of Ca^2+^, resulting in JNK phosphorylation, activation of the p53 apoptosis signal pathway, upregulation of the expression of pro-apoptotic factors (PUMA and Caspase3), and the induction of apoptosis [20]. Studies report that mitochondria also play a role in apoptosis [48]. Khan et al. observed that mitochondria induce the production of cytochrome C (CytoC) by p53-activated BAX. BAX is a member of the Bcl-2 family of pro-apoptotic proteins which promotes the expression of Caspase9, as well as the expression of Caspase3, ultimately inducing cell apoptosis [49]. Notably, FB_1_ may induce apoptosis through the Fas/FasL pathway. FB_1_ promotes dysfunction of the Fas receptor and activation of the caspase8 pathway inducing Caspase3 expression and resulting in cell apoptosis [50,51]. Kim et al. reported that oxidative stress mediates the JNK pathway through the effects on the endoplasmic reticulum, as well as promotes interaction between Bcl-2 and BECN-1 to release BECN1. BECN1 is a key regulator of autophagy. BECN1 modulates expression of the autophagy-associated protein LC3-II/I to induce autophagy and promotes autophagy through the endoplasmic reticulum-mediated expression of Inositol-requiring enzyme-1-α (IRE1-α), PERK-induced ATG5, ATG7, and LC3-II/I [20]. Notably, exposure to FB_1_ upregulates the expression of AMP-dependent protein kinase (AMPK) and downregulates expression of mammalian target of rapamycin (mTOR) by mediating endoplasmic reticulum stress, thus inducing autophagy [38]. Moreover, tumor necrosis factor-alpha (TNF-α) plays an important role in the toxicity induced by FB_1_. He et al. reported that the activity of TNF-α and expression level of TNF-α mRNA in heart and lung tissues of mice increased after a subcutaneous administration of 2.25 mg/kg-BW FB_1_ to male and female BALB/c mice for 5 days [52]. KÓCSÓ et al. reported similar findings that FB_1_ upregulates TNF-α mRNA expression and increases the activity of TNF-α [53]. Chen et al. reported that FB_1_ upregulated the expression of TNF-α mRNA in PK-15 cells and induced apoptosis in porcine kidney cells PK-15, indicating that TNF-α can be used as a biomarker for FB_1_ exposure in vivo [54]. Régnier et al. observed that administration of 10 mg/kg FB_1_ through diet upregulated the expression of NF-κB and Interleukin-8 in the liver and jejunum. Notably, NF-κB is an important target in the TNF signal pathway, implying that TNF-α may play a role in the toxicity induced by FB_1_ [55]. The mechanism of fumonisin toxicity is presented in Figure 4.

## 3. Detoxification of Fumonisins Using Biological Antioxidants

**Figure 5:**
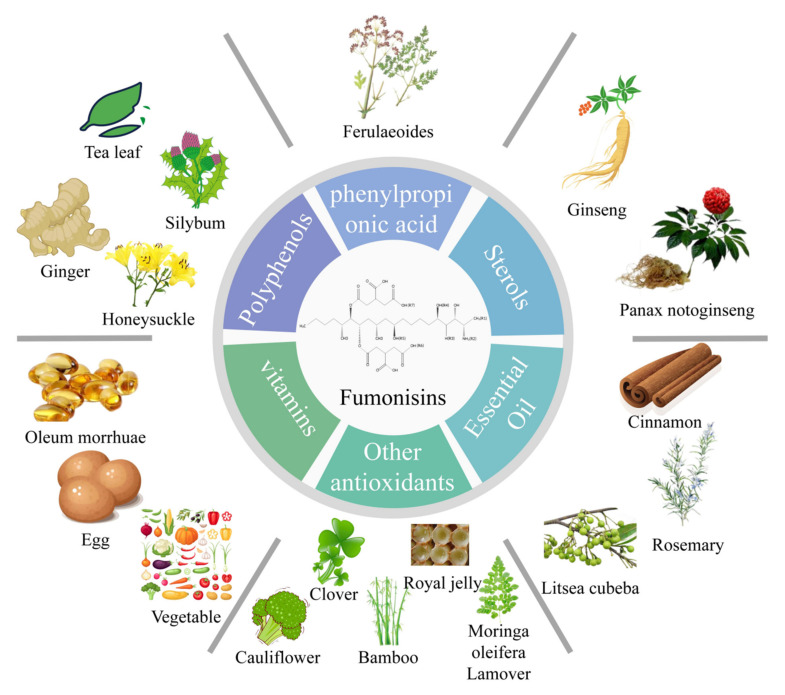
Summary diagram of biological antioxidants. Antioxidants are widely found in vegetables and other green plants, indicating that eating more green leafy vegetables may have a protective effect on fumonisin-induced toxicity.

The biological detoxification mechanism of fumonisins is mainly through the antioxidant neutralization of reactive oxygen species caused by oxidative stress, thus reducing the effects of fumonisins mediated through oxidative stress. Biological antioxidants include polyphenols, sterols, phenyl propionic acids, fat-soluble and water-soluble substances, plant essential oils, and other antioxidants (Figure 5).

### 3.1. Polyphenols

**Figure 6:**
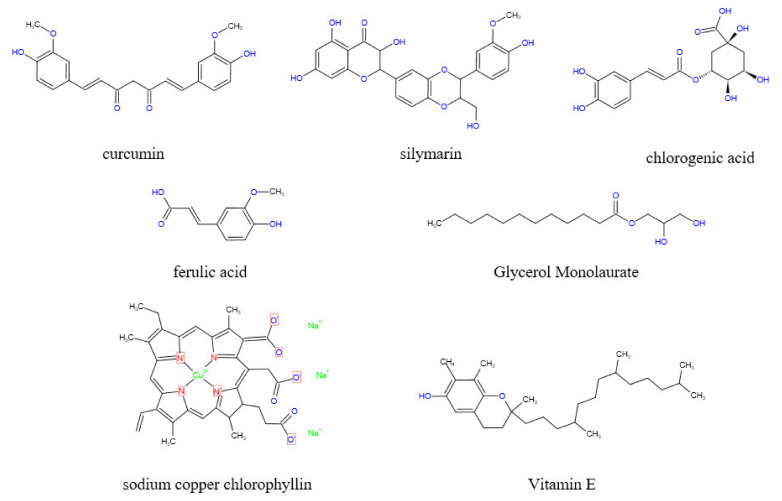
Active components of antioxidants. Hydroxyl groups are widely found in antioxidants and have a strong reducing ability; thus, they can neutralize excessive reactive oxygen species produced under oxidative stress and achieve an antioxidant effect.

Curcumin is a natural polyphenolic compound extracted from the rhizome of Zingiberaceae and other plants. (Figure 6) [56]. Notably, curcumin increases ceramide concentration by stimulating de novo synthesis of ceramide, activating neutral sphingomyelinase and inhibiting the activity of sphingomyelin synthase [57]. Lloyd-Evans et al. observed that curcumin reduces the intracellular accumulation of So, sphingomyelin, glycosphingolipids, and cholesterol by restoring the intracellular calcium content. These characteristics are the main features of Niemann-PicktypeC1 disease [58], as well as features for fumonisin poisoning. Feeding chicks with curcumin nanocapsules supplemented with 600 mg/kg fumonisin and 10 mg/kg curcumin, showed protecting protective effects to the liver and an antioxidant effect, as well as reducing the level of thiobarbituric acid active substance in ROS and improving the weight gain of chicks compared with the control group [59]. Moreover, curcumin reduces PK-15 death in vitro. Administration of curcumin PK-15 cells pretreated with 50 μM FB_1_ showed an increase in the cell survival rate from 53.7% to 77% and a decrease in the intracellular ROS content from 97.4% to 75.5% [60]. Silymarin (SIL) is also a polyphenol with a similar effect to that of curcumin (Figure 6). Sozmen et al. reported that SIL significantly reduced hepatocyte apoptosis (*p* < 0.0001) and upregulated the expression of Caspase-8 and TNF-α (*p* < 0.0001) in BALB/c mice treated with 100 mg/kg FB_1_ as well as 1.5 mg/kg SIL in vivo [61]. Furthermore, Ledur et al. observed that the administration of 50 μM FB_1_ to PK-15 cells pretreated with 2.5 μM SIL increased the cell survival rate from 53.7% to 89.2% and decreased the intracellular ROS content from 97.4% to 34.2% [60]. Moreover, Marnewick et al. reported that tea polyphenols alleviate hepatotoxicity induced by FB_1._ For instance, the administration in male Fischer rats of 250 mg/kg FB_1_ and aqueous extracts of rooibos (*Aspalathus linearis*), honeybush (*Cyclopia intermedia*), herbal, and green and black (*Camellia sinensis*) teas before and after fermentation showed a significant increase in the scavenging ability of mouse liver cells to free radicals. In addition, fermented herbal teas and unfermented honeybush significantly reduced liver lipid peroxidation induced by FB_1_. Moreover, the three tea extracts improved the activities of CAT, GPx, and GR at varying degrees [62]. Chlorogenic acid also has an inhibitory effect on fumonisin-producing strains (Figure 6). Chlorogenic acid is a common dietary polyphenol with significant bioactivity. The inhibition rate of fumonisin-producing strains after administration of chlorogenic acid was up to 70% [63].

### 3.2. Sterols

Hassan et al. explored the protective effect of ginseng extract (PGE) on mice exposed to FB_1_, as PGE contains a lot of sterols such as ginsenosides. The findings indicated that PGE reduced fragmentation of DNA in the liver and kidney after the administration of 20 mg/kg-BW of PGE and 100 μg/kg-BW FB_1_ to male mice at the same time. Moreover, PGE alleviated LP changes in the liver and kidney, increased GSH level, and upregulated GPx, SOD1, and CAT mRNA expression. In addition, the GPx, SOD1, and CAT mRNA expression levels of mice in the PGE group treated with 20 mg/kg-BW of FB_1_ were significantly higher relative to the expression levels of mice in the blank control group [64]. Additionally, Abdel-Wahhab et al. explored the effect of red ginseng on FB_1_ toxicity in Sprague-Dawley rats and reported consistent findings [65]. The root extract of *Panax notoginseng* has an inhibitory effect on the carcinogenicity of FB_1_. Takao et al. administered FB_1_ and acetone to female SENCAR mice through a skin smear to stimulate papilloma formation. The treatment group was administered with *Panax notoginseng* acetone extract 1 h before each administration of FB_1_. The findings showed that 100% of the mice in the control group developed papilloma after 12 weeks of FB_1_ and acetone skin smearing, whereas only about 20% and 50% of the mice in the treatment group developed papilloma after 12 and 15 weeks, respectively [66]. Moreover, daily consumption of ginseng may have a preventive or detoxifying effect on fumonisin toxicity.

### 3.3. Phenylpropionic Acids

Ferulic acid is a phenyl propionic acid compound derived from Ferula feruloides (Steudel) Korovin and other plants (Figure 6). Ferulic acid at 10–25 mM significantly decreased the growth rate of *Fusarium oxysporum* compared with the control group (*p* < 0.001). In addition, fumonisin production was inhibited to a certain extent [67]. Ferulic acid can be extracted from cheap agricultural by-products, therefore, the extraction of ferulic acid from low-cost agricultural by-products can be an important source in controlling the production of fumonisins in plants [68,69].

### 3.4. Vitamins

Vitamin E is an important antioxidant (Figure 6). Pretreatment of mice with 25 µM vitamin E (tocopherol) for 24 h before 18 µM FB_1_ treatment significantly reduces FB_1_-induced DNA damage and apoptosis [70,71]. In addition, vitamin E can be combined with selenium, CoQ10, and L-carnitine to prepare a compound with synergistic effects. In a previous study, mice were pretreated with vitamin E (30 IU/kg), selenium (1 mg/kg), CoQ10 (30 mg/kg), and L-L-carnitine (2.8 mg/kg), then intravenously administered with 1.55 mg/kg-BW FB_1_. The results indicated that a combination of these antioxidants alleviated DNA damage and increased the activities of aspartate aminotransferase and alanine aminotransferase by 18% and 18%, respectively, compared with the level of mice not exposed to FB_1_ [33]. Oginni et al. administered juvenile catfish with vitamin E and vitamin C at the same time and observed that the decrease in nutrient content in juvenile catfish induced by FB_1_ was improved. Notably, the crude protein content in juvenile catfish was higher compared with that of the FB_1_ group (*p* < 0.05) [72]. Furthermore, folic acid has a protective effect on cytotoxicity induced by fumonisins. Sadler et al. reported that folic acid reduced the toxic effect of FB_1_ on mouse embryos and improved the growth of mouse embryos after culturing embryos with a mixture of 10 mM folic acid and 2 µM FB_1_, indicating that folic acid improves the toxic effect of fumonisins, however, the change was not significant [73].

### 3.5. Essential Oil

Essential oils are unique aromatic substances extracted from plants, mainly containing alcohols, aldehydes, phenols, acetones, terpenes, and other volatile secondary metabolites synthesized by plants [74,75]. Several types of essential oils such as *Litsea cubeba*, cinnamon, and ginger have been reported, and most have an inhibitory effect on bacterial growth. Pante et al. conducted an in vitro experiment and reported that *Litsea cubeba* essential oil inhibited mycelial development of *Furium verticillioides* and synthesis of FB_1_ and FB_2_. The minimum inhibitory concentration of *Fusarium verticillioides* was 125 µg/mL and the inhibitory effect was dose dependent. The antioxidant effect of *Litsea cubeba* essential oil was evaluated by DPPH and ABTS methods, showing excellent antioxidant activity [76]. Bomfim et al. reported that *Rosmarinus officinalis* L. essential oil (REO) had a similar effect. Administration of 300 µg/mL REO caused significant morphological changes such as bacterial cell wall rupture and cell content flow out in a dose-dependent manner [77]. In addition, *Zingiber officinale* essential oil (GEO) inhibits the growth of fumonisin-producing bacteria and fumonisin production. Notably, administration of 2000 µg/mL GEO and 4000 µg/mL GEO significantly inhibits the production of FB_1_ and FB_2_. The inhibition rates of ergosterol biosynthesis after administration of 4000 µg/mL and 5000 µg/mL GEO were 57% and 100%, respectively [78]. Ergosterol modulates the activity of several membrane binding enzymes [79], and the reduction of ergosterol activity could result in membrane synthesis disorders, thus exhibiting a bacteriostatic effect. Castro et al. reported similar results with minimum inhibitory concentrations of *Cinnamomum zeylanicum* and *Cymbopogon martinii* essential oils to *Fusarium verticillioides* at 250, 250, and 500 µg/mL, respectively [80]. Plant essential oils inhibit the growth of fumonisin-producing bacteria and fumonisin production, as well as reduce or prevent toxicity caused by fumonisins. Essential oils have a strong smell and react with some drugs, thus, embedding technology is commonly used to embed essential oils. Cinnamon essential oil embedded with whey protein effectively improved the serum levels of ALT, AST, ALP, Urea, and Uric acid, and restored the normal levels in male Sprague-Dawley male rats treated with 100 mg/kg-BW FB_1._ Furthermore, testosterone levels in rats were restored to normal values thus reducing reproductive toxicity. Lipid peroxidation and tumor marker TNF-α in liver and kidney tissues were improved to some extent but were not restored to normal levels [74]. Studies report that the cinnamon extract glycerol monolaurate (GML) (Figure 6) has similar effects. The levels of serum triglyceride, globulin, cholesterol, liver lipid peroxidation, SOD, and serum reactive oxygen species were restored to normal or below normal levels after chicks were fed with 400 µg/kg fumonisins and GML coated with 8 mg/kg nanomaterials. However, the body weight of chicks was not improved indicating that GML does not reduce the oxidative stress caused by fumonisins to a minimum. However, it alleviates oxidative stress caused by fumonisins and enhances the activity of glutathione S-transferase which is the enzyme responsible for liver detoxification [81].

### 3.6. Other Antioxidants

In addition to the above-mentioned antioxidants, several other types of antioxidants have been reported in previous studies. Domijan et al. reported that sodium copper chlorophyllin (CHL) had a protective effect on FB_1_-induced cell and DNA damage after administration of 100 µg/mL (CHL) (Figure 6) in combination with 20 µg/mL of FB_1_. Oxidative stress is the main cause of DNA damage caused by FB_1_, thus CHL indirectly prevented FB_1_-induced cell death, DNA damage, and possible carcinogenesis by preventing oxidative stress [82]. Zhao et al. conducted a study whereby indole glucosinolates (IGS) (Figure 5) were infiltrated into wild-type Col-0 plants followed by a 10 µM FB_1_ solution into the wild-type IGS plants and compared the results with the administration of only the FB_1_ solution. The findings showed that IGS inhibited FB_1_-induced apoptosis. IGS decomposition products produced through the action of β-glucosinolase effectively reduce the accumulation of ROS, increase the activity of antioxidant enzymes, and improve ROS scavenging ability, thus reducing FB_1_-induced oxidative stress and apoptosis [83]. CHL and IGS are widely distributed in green leafy vegetables, thus eating more green leafy vegetables may have a preventive effect on fumonisins toxicity.

In addition to single-component antioxidants, several compound antioxidants have been reported. Hassan et al. observed that all biochemical and cytogenetic test parameters and histological images of liver tissue were significantly improved after feeding mice with an ethanol extract of *Aquilegia vulgari*s L. at 10 mg/kg-BW and 200 mg/kg voronisin [84]. Gbore et al. reported that the food intake of female rabbits approached the normal level after administration of Moringa leaf meal (MLM) in combination with FB_1_ and the effect of MLM was dose dependent. The antioxidant effect of MLM improved the adverse effects of FB_1_ on nutrient utilization and growth performance of female rabbits. Notably, MLM is a cheaper alternative compared with commercial antioxidants. MLM can be used as an antidote in traditional feed to reduce the harmful effects of FB_1_ on domestic animal production [85].

Moreover, insect products have antioxidant effects. Several honeybee products are potential sources of natural antioxidants and can counteract the effects of oxidative stress caused by various diseases [86]. Royal jelly (RJ) contains several bioactive substances and phenolic compounds, mainly comprising flavonoids and fenac, and has antioxidant activities. Liver and kidney indexes were significantly improved when male Sprague-Dawley rats were administered with a combination of 200 mg/kg fumonisins and 150 mg/kg-BW RJ compared with the levels in mice fed with FB_1_ alone. Liver and kidney indexes were also restored to normal levels, indicating that RJ has a protective effect on fumonisin toxicity. Notably, the protective effect was dose dependent [87].

## 4. Antagonistic Microorganisms and Degradation Mechanism of Fumonisins

The use of microbial control has become a research hotspot and has a wide research prospect in the field of biological control of fumonisin-producing strains and fumonisin toxin degradation. Studies report that microorganisms produce metabolites or the microorganisms themselves have potential active substances that inhibit fumonisin production. Microorganisms including lactic acid bacteria, yeasts, Klebsiella, Bacillus, sphingomonas, and other microorganisms, exert inhibitory effects on fumonisin-producing strains and fumonisin activity.

### 4.1. Microbial Removal of Fumonisins and Its Mechanism

**Figure 7:**
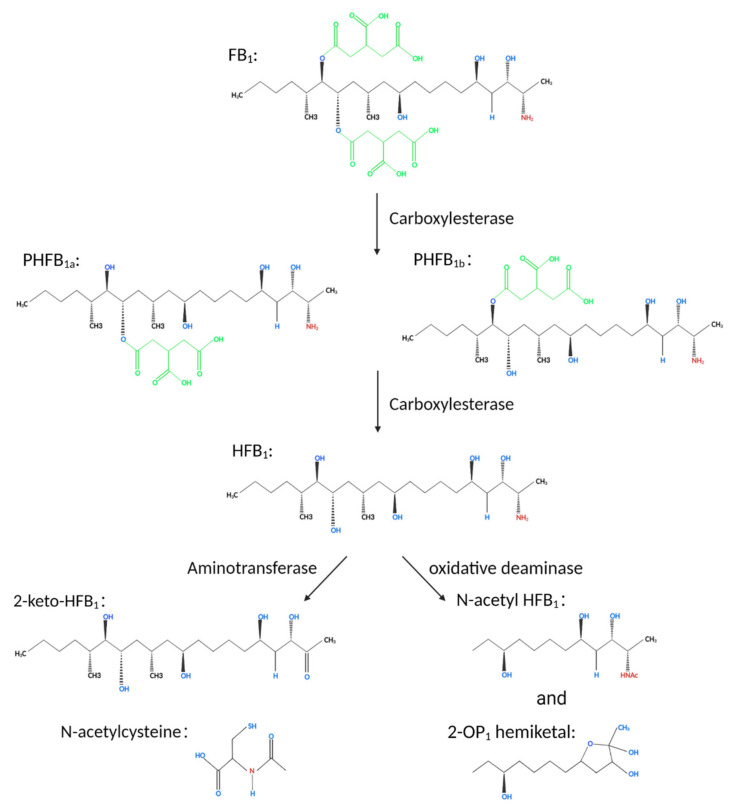
A schematic representation of the degradation process of FB_1_. Degradation of FB_1_ occurs through a three-step reaction. The structure of FB_1_ is gradually disintegrated after the completion of the reaction and is converted from toxic to low-toxic or non-toxic compounds.

Fumonisins are a chemically stable diester compound with a structure composed of different polyhydroalcohols and propanotricarboxylic acids. The skeleton is a fat chain comprising 20 carbon atoms and two identical lipid bond side chains connected to carbon 14 and 15 atoms. The degradation of fumonisins by microorganisms that use fumonisins as the only carbon source can be divided into two steps. In the case of FB_1_, the tricarboxylic acid groups on carbon 14 and 15 are cleaved under the action of carboxylesterase to form hydrolyzed FB_1_ (HFB_1_). The toxicity of HFB_1_ is significantly lower compared with that of FB_1_. Further, HFB_1_ is degraded to 2-keto-HFB_1_ under the action of a transaminase enzyme [88]. Further studies indicate that FB_1_ is first degraded into partially hydrolyzed FB_1_ (PHFB_1__a_ or PHFB_1__b_) under the action of carboxylesterase. These two metabolites are formed through the cleavage of tricarboxylic acid groups on carbon 14 or 15 of FB_1._ The metabolites further react to form HFB_1_, which further reacts under the action of transaminase enzyme to form 2-keto-HFB_1_ as well as N-acetyl HFB_1_ or 2-OP_1_ semi-Ketal under the action of oxidative deaminase enzyme (Figure 7) [89,90,91].

The main microorganism that degrades fumonisins is sphingomonas which is widely distributed in aquatic and terrestrial environments. Sphingomonas is widely used in mycotoxin degradation. Studies report two key genes implicated in fumonisin degradation in sphingomonas namely, MTA144 which encodes transaminase and carboxylesterase. Notably, MTA144 degradation does not depend on the aerobic environment, thus it can be carried out in an anaerobic environment [89]. Moreover, the ATCC 55552 strain contains a gene that encodes a fumonisin degradation enzyme. The gene can be highly expressed in *Escherichia coli* and the recombinant enzyme has deamination activity in the presence of α-ketopyruvate and a pyridoxal phosphate coenzyme, thus it can deaminate HFB_1_. Studies should explore the genetic basis of HFB_1_ deamination in bacterial ATCC 55552 to fully understand the catabolism of FB_1_ [90].

Zhao et al. isolated a group of bacteria named SAAS79 which efficiently degraded fumonisins from mushroom residue and were mainly composed of *Pseudomonas*, *Delftia*, *Sphingobacterium*, *Achromobacter,* and other species, whereby *Pseudomonas* played a significant role in FB_1_ degradation. The findings showed that the degradation rate of 10 µg/mL FB_1_ was more than 90% under a pH of 5~7 and a temperature of 28~35 °C for 24 h. Moreover, the degradation rate of intracellular enzymes on approximately 10 µg/mL FB_1_ was about 90% after incubation for 3 h. The flora exhibited a high degradation efficiency, mild action conditions, and a wide degradation temperature range, and could degrade FB_1_ at a temperature ranging from 20 to 50 °C. Therefore, it has a high potential in the feed and food industry for the effective removal of FB_1_ [92]. Tuppia et al. explored the effect of nine strains on degrading FB_1_ from silage grain with high moisture content. The findings showed that *Lactobacillus brevis* N195 and N197 had the highest degradation effects on FB_1_, with degradation rates of 33% and 30% in vitro, respectively. Notably, the degradation rate of FB_1_ in silage grains was more than 90%, indicating that the degradation process was accompanied by adsorption, however, the specific detoxification mechanism should be explored further [93]. Benedetti et al. reported a strain that could degrade FB_1_ in soil. The NCB 1492 strain that degraded FB_1_ was isolated from the culture medium by enrichment culture. Notably, 16SrRNA analysis showed that the strain was a member of the Delftia or Comamonas family. TLC analysis showed that the NCB 1492 strain degraded FB_1_ 24 h after culturing it in phosphate buffer saltwater (PBS) supplemented with 0.5 mg/mL FB_1._ FB_1_ spots were not detected after culturing the strain in phosphate PBS with 0.5 mg/mL FB_1._ No fluorescence peak was observed in HPLC analysis after incubation at 25 °C for 2 h, indicating that the NCB 1492 strain degraded fumonisins [94].

Genetically engineered bacteria have a better degradation activity of fumonisin compared with naturally occurring microorganisms. Biomin company constructed *Komagataella Pastoris* engineered yeast through genetic engineering technology. This strain produces fumonisin lipase named FUMzyme^®^ (Biomin company, Wuxi, China). FUMzyme^®^ is used in various poultry feeds. The lowest dose of 15 enzyme activity units (U)/kg and the highest dose of 300 U/kg showed the best activity. FB_1_ in poultry feces decreased significantly (*p* < 0.001) when 250 U/kg of FUMzyme^®^ was added to the feed. The F_1_ in poultry feces also decreased significantly when FUMzyme^®^ was added to feed (*p* < 0.05). In addition, the ratio of Sa/So in the liver and serum in poultry was lower relative to that in the control group [95]. The bacteria constructed by genetic engineering have several advantages such as a high concentration, high yield, and controllable metabolism. Moreover, the background of basic biology and molecular genetics is fully elucidated, thus biological safety is guaranteed. These findings indicate that the construction of genetically engineered bacteria has high potential in microbial removal of fumonisins in the future.

### 4.2. Microbial Adsorption of Fumonisins

In addition to degradation, some microorganisms can adsorb fumonisins. The adsorption effect of these microorganisms is mainly affected by temperature, pH, and bacterial structure. High-performance liquid chromatography and liquid chromatography/mass spectrometry (LC/MS) are commonly used to detect the degradation and adsorption of mycotoxins by strains. The removal of mycotoxins by strains was verified by detecting the presence of fumonisin degradation products in co-cultures. Lactic acid bacteria are widely used as probiotics owing to their large specific surface area and complex surface structure. Lactic acid bacteria are the main bacteria used for microbial adsorption and removal of fumonisins. Furthermore, they have a broad application prospect in the control of mycotoxins.

Deepthi et al. explored the effect of feeding broilers with feed containing 1 mL (10^9^ CFU/mL) *Lactobacillus plantarum* MYS6 and 200 mg/kg FB_1_ daily for 42 days. The findings showed that *Lactobacillus plantarum* MYS6 restored the levels of serum parameters such as SGOT, SGPT, creatinine, cholesterol, and triglyceride to the level of the blank control group. Moreover, this strain effectively removed ROS and H_2_O_2_ from serum and liver tissue compared with the FB_1_ group [96]. *Lactobacillus plantarum* MYS6 and its extracellular products were added to the feed, and the results indicated that the content of fumonisins in the feed decreased from the original 0.870 mg/kg to 0.505 mg/kg and 0.3125 mg/kg, after co-culture of *Lact*obacillus plantarum MYS6 and FB_1_ after 2 h and 4 h, respectively, in vitro. The removal rates of FB_1_ were 32.9% and 61.7% at 2 h and 4 h, respectively. LC/MS analysis showed that the corresponding peaks of fumonisin degradation products were not present after 2 and 4 h of incubation, implying that the mechanism of fumonisin removal may be physical adsorption [97]. A previous study reported that *Lactobacillus plantarum* ZJ8 had a high binding rate to fumonisins in an acidic environment. The binding rate of the cell wall of ZJ8 strain to FB_1_ and FB_2_ was 96.8% and 100%, respectively, at pH 4 [98]. In addition, *Lactobacillus plantarum* B7 and *Lactobacillus pentosus* X8 exhibited a high scavenging effect on fumonisins, and the binding rate was dependent on the structural integrity of bacterial cell wall peptidoglycan. High peptidoglycan integrity was correlated with high binding efficiency. When peptidoglycan was removed, the removal rate of fumonisin was greatly reduced, which was speculated to be the mechanism of adsorption. The binding rates of *Lactobacillus plantarum* B7 and *Lactobacillus pentosus* X8 to FB_1_ and FB_2_ were 52.9% and 58.0%, respectively [99]. In addition to peptidoglycan integrity, acid resistance and protease resistance play an important role in mycotoxin adsorption. Ezdini et al. reported that *Lactobacillus* par*asitum* BEJ01 isolated from Tunisian butter had a strong tolerance to acid as well as to pepsin. In a previous study, male Balb/c mice were fed with 100 μg/kg FB_1_ and 2 × 10^9^ CFU/mL BEJ01 for 10 days and stopped feeding for 48 h wherein the biochemical indexes of the mice were detected. The results showed that the content of MDA and the ability of binding catalase in the liver and kidney tissue were significantly lower relative to those in the FB_1_ group whereas the levels were similar to those of the normal feeding group [100]. Abbès et al. explored the detoxification mechanism of *Lactobacillus parasitum* BEJ01. Their findings indicated that *Lactobacillus parasitum* BEJ01 adsorbed 67.5% of FB_1_ in PBS in 12 h, and the adsorption rate was time dependent. The adsorption rate of FB_1_ reached 88.9% at 24 h and the adsorption capacity of living bacteria was significantly higher compared with that of dead bacteria. The heating and sterilization of bacteria reduced the adsorption capacity of FB_1_ to 25.5% [101]. Therefore, the survival of bacteria should be ensured when adsorbing toxins.

Moreover, *Saccharomyces cerevisiae* is used in the scavenging of fumonisins. Armando et al. used the Plackett-Burman screening design and central combinatorial design to screen and evaluate the ability of the *Saccharomyces cerevisiae* RC016 strain on the removal of FB_1_ in vitro. The findings showed that the scavenging ability of *Saccharomyces cerevisiae* RC016 reached 78.66% with a concentration of FB_1_ at 50 μg/mL and the binding ability decreased with the decrease in FB_1_ concentration [102]. This implies that microbial adsorption is an important mechanism in fumonisin removal.

## 5. Conclusions and Perspectives

Fumonisin contamination in food and feed has posed a serious threat to economic development and public health security. How to effectively control and degrade fumonisins in food and feed has become an urgent scientific problem for human health. Chemical treatments such as alkali treatment, ozone treatment, ammonia treatment, monomethylamine, and calcium hydroxide treatment may be effective. However, the residues of these chemicals in food and feed cannot be solved economically and effectively, and the application of chemical control methods is severely limited by the problems of fungal resistance, chemical residues, and environmental pollution. Meanwhile, mycotoxin adsorbents commonly used in practical production not only adsorb mycotoxin but also adsorb small molecular substances in feed, such as biotin. In addition, the adsorption capacity and adsorption effect of various adsorbents for different toxins are different, and the combination of multiple adsorbents at the same time could achieve the desired adsorption effect, though the superimposed effect needs to be demonstrated through further experiments. Biological antioxidants have the advantages of good biodegradability, low residue, and environmental friendliness and can replace chemical synthesis agents in antibacterial and mildew control. As an important source of new green mildew inhibition agents, biological antioxidants have great development potential and broad application prospects.

In all kinds of fumonisin detoxification technologies, biological detoxification has the advantages of a good detoxification effect, no residue, and small nutritional loss. Degradation of fumonisins into less toxic substances by using proteins or enzymes produced by microorganisms and the screening of microorganisms that have an antagonistic effect on virulence strains will be the focus of future research in the field of fumonisin control. It should be noted that there are still many problems to be solved in order to realize the scale application of biological detoxification technology. The degradation of fumonisins by microorganisms is affected by enzyme and microbial concentration as well as environmental parameters. Due to the complex separation and purification process of microbial enzymes and the harsh conditions of enzyme action, there are few studies on mycotoxin recombinant enzymes and their practical production and application. Therefore, screening microorganisms that can effectively remove many kinds of fumonisins and isolating and purifying the compound detoxification enzymes secreted by them are the keys to successfully controlling fumonisins. It is also an important breakthrough point and development direction in the field of fumonisin biological detoxification research to find and screen bacteria that can degrade fumonisins, to study the characteristics of the extracellular detoxification enzyme produced by bacteria, and to clone and express the detoxification enzyme gene. In the future, modern molecular biology methods and genetic engineering methods should be considered to clone and express highly active detoxifying enzyme genes so as to achieve large-scale application as an independent or combined technology.

## Figures and Tables

**Table 1:** Fumonisins and their structural analogs [10].

Fumonisins	R1	R2	R3	R4	R5	R6	R7
FB_1_	CH3	NH2	H	OH	OH	TCA	TCA
Iso-FB_1_	CH3	NH2	OH	H	OH	TCA	TCA
PHFB_1a_	CH3	NH2	H	OH	OH	OH	TCA
PHFB_1b_	CH3	NH2	H	OH	OH	TCA	OH
HFB_1_	CH3	NH2	H	OH	OH	OH	OH
FB_2_	CH3	NH2	H	OH	H	TCA	TCA
FB_3_	CH3	NH2	H	H	OH	TCA	TCA
FB_4_	CH3	NH2	H	H	H	TCA	TCA
FBK_1_	CH3	NH2	H	H	OH	TCA	=O
FA_1_	CH3	NHCOCH3	H	OH	OH	TCA	TCA
FA_2_	CH3	NHCOCH3	H	OH	H	TCA	TCA
FA_3_	CH3	NHCOCH3	H	H	OH	TCA	TCA
PHFA_3a_	CH3	NHCOCH3	H	H	OH	OH	TCA
PHFA_3b_	CH3	NHCOCH3	H	H	OH	TCA	OH
HFA_3_	CH3	NHCOCH3	H	H	OH	OH	OH
FAK_1_	CH3	NHCOCH3	H	OH	OH	TCA	=O
FC_1_	H	NH2	H	OH	OH	TCA	TCA
N-acetyl-FC_1_	H	NHCOCH3	H	OH	OH	TCA	TCA
Iso-FC_1_	H	NH2	OH	H	OH	TCA	TCA
N-acetyl-iso-FC_1_	H	NHCOCH3	OH	H	OH	TCA	TCA
OH-FC_1_	H	NH2	OH	OH	OH	TCA	TCA
N-acetyl-OH-FC_1_	H	NHCOCH3	OH	OH	OH	TCA	TCA
FC_3_	H	NH2	H	H	OH	TCA	TCA
FC_4_	H	NH2	H	H	H	TCA	TCA
FP_1_	CH3	3HP	H	OH	OH	TCA	TCA
FP_2_	CH3	3HP	H	OH	H	TCA	TCA
FP_3_	CH3	3HP	H	H	OH	TCA	TCA

## Data Availability

Not applicable.

## Abbreviations

FB_1_	fumonisins B_1_
FB_2_	fumonisins B_2_
BW	body weight
So	sphingosine
Sa	sphinganine
So-1-p	sphingosine-1-phosphate
Sa-1-p	sphinganin-1-phosphate
SPT	serine palmitoyltransfe
3-KeSa	3-keto-sphinganine
3-KR	3-keto reductase
dhCerS	dihydroceramide synthetase
CerS	ceramide synthetase
Cer	ceramide
Ser	serine
dhDES	dihydroceramide dehydrogenase
C1P	ceramide-1-phosphate
CERK	ceramide kinase
C1PP	C1P phosphatase
CDases	ceramidase
SoKs	sphingosine kinase
So-1-pP	sphingosine-1-phosphate phosphatase
PE	phosphoethanolamine
HE	hexadecanal
So-1-pL	So-1-p lyase
SMases	sphingomyelinase
SMSs	sphingomyelin synthase
GlySM	Glycosphingolipids
GBA	glucerebrosidase
GCS	galactosyl-ceramide synthase
GSH	glutathione
GPx	glutathione peroxidase
SOD	superoxide dismutase
GR	glutathione reductase
CAT	catalase
MDA	malondialdehyde
MAPK	mitogen-activated protein kinase
PKC	protein kinase C
JNK	c-Jun N-terminal kinase
IRE1-α	Inositol-requiring enzyme-1-α
AMPK	AMP-dependent protein kinase
mTOR	mammalian target of rapamycin
TNF-α	tumor necrosis factor alpha
CytoC	cytochrome C
SIL	silymarin
PGE	protective effect of ginseng extract
REO	*Rosmarinus officinalis* L. essential oil
GML	glycerol monolaurate
CHL	chlorophyllin
IGS	indoleglucosinolates
MLM	moringa leaf meal
RJ	royal jelly
HFB_1_	hydrolyzed FB_1_
PHFB_1_	partially hydrolysed FB_1_
PBS	phosphate buffer saltwater
